# Functional outcome after perineal stapled prolapse resection for external rectal prolapse

**DOI:** 10.1186/1471-2482-10-9

**Published:** 2010-03-08

**Authors:** Franc H Hetzer, Amir H Roushan, Katja Wolf, Ulrich Beutner, Jan Borovicka, Jochen Lange, Lukas Marti

**Affiliations:** 1 Department of Surgery, Cantonal Hospital, St. Gallen, CH-9007 St. Gallen Switzerland; 2 Division of Gastroenterology and Hepatology, Cantonal Hospital, CH-9007 St. Gallen, Switzerland

## Abstract

**Background:**

A new surgical technique, the Perineal Stapled Prolapse resection (PSP) for external rectal prolapse was introduced in a feasibility study in 2008. This study now presents the first results of a larger patient group with functional outcome in a mid-term follow-up.

**Methods:**

From December 2007 to April 2009 PSP was performed by the same surgeon team on patients with external rectal prolapse. The prolapse was completely pulled out and then axially cut open with a linear stapler at three and nine o'clock in lithotomy position. Finally, the prolapse was resected stepwise with the curved Contour^® ^Transtar™ stapler at the prolapse's uptake. Perioperative morbidity and functional outcome were prospectively measured by appropriate scores.

**Results:**

32 patients participated in the study; median age was 80 years (range 26-93). No intraoperative complications and 6.3% minor postoperative complications occurred. Median operation time was 30 minutes (15-65), hospital stay 5 days (2-19). Functional outcome data were available in 31 of the patients after a median follow-up of 6 months (4-22). Preoperative severe faecal incontinence disappeared postoperatively in 90% of patients with a reduction of the median Wexner score from 16 (4-20) to 1 (0-14) (*P *< 0.0001). No new incidence of constipation was reported.

**Conclusions:**

The PSP is an elegant, fast and safe procedure, with good functional results.

**Trial registration:**

ISRCTN68491191

## Background

In 2007, we introduced a new surgical technique for external rectal prolapse, the Perineal Stapled Prolapse resection (PSP), and published the results of an initial feasibility study the following year [1]. The PSP was completed in 14 out of 15 patients with no intraoperative complications. Apart from two patients with postoperative bleeding from the stapler line, which required transanal oversewing, no major complications occurred. No early recurrence of the prolapse was found during the postoperative follow-up of 3 months. We concluded that the new procedure is safe and were encouraged use of the new technique on more patients.

Compared to established perineal procedures such as Delorme's procedure, perineal rectosigmoidectomy [2] or perineal rectosigmoidectomy with levatorplasty [3], the main advantage of the PSP is the shorter operating time [1]. In contrast to the modified perineal rectosigmoidectomy procedure with a stapled anastomosis using a circular stapler, the PSP creates a larger median circumference with less postoperative capacity reduction and less anastomotic stenosis [1]. On the other hand, the higher cost for the stapler had to be calculated and the functional outcome is pending.

Over time we refined and improved the initial technique and performed the PSP on a wider range of patients. The aim of this study is to present the functional outcome and the early follow-up from a larger number of patients.

## Methods

From December 2007 to April 2009 all patients with an external rectal prolapse were evaluated for PSP. We discussed the advantages and disadvantages of the PSP method with every patient and decided together on the surgical technique. For frail patients with severe co-morbidities and short life expectancy, we recommended the perineal technique in accordance with published recommendations [4,5]. The usual preoperative examinations (full history, complete physical examination, electrocardiogram, chest x-ray and assessment of the coagulation and haemogram) were performed. Then the patients underwent PSP with no bowel preparation. Before and after the procedure patients were interviewed regarding their bowel function to obtain the Wexner score. Twenty-one patients preoperatively complained of severe incontinence (Wexner score ≥ 12) and 12 patients of constipation, according to Rome II criteria, respectively [6]. A significant postoperative improvement in continence was arbitrary, defined as a postoperative halving of the preoperative Wexner score.

At the start of the operation, a single dose antibiotic prophylaxis with a combination of a cephalosporin (Mandokef^® ^2 grams) and metronidazole (Flagyl^® ^500 milligrams) was administered intravenously. The same surgeon (FHH) was present at every operation either performing the procedure himself or supervising colleagues. No patient from the previous feasibility study [1] was included in this study. All complications were recorded prospectively during the hospital stay and follow-up and classified as proposed by Dindo D. et al [7]. Follow-up visits were performed one month after the operation (n= 32 patients) and a telephone interview for the functional outcome after 2 (N = 32) and for the assessment of recurrence after 6 (n = 18) and 12 (n = 8) months.

The cantonal ethics committee ("Ethikkommission des Kantons St. Gallen") approved rectal prolapse resection with the Contour^® ^Transtar™ stapler and the prospective data collection and the trial was registered on http://www.controlled-trials.com under the number ISRCTN68491191. From all patients written consent was obtained for surgery, for anonymous data collection and for publication of those findings and images taken.

### Surgical Technique

**Figure 1:**
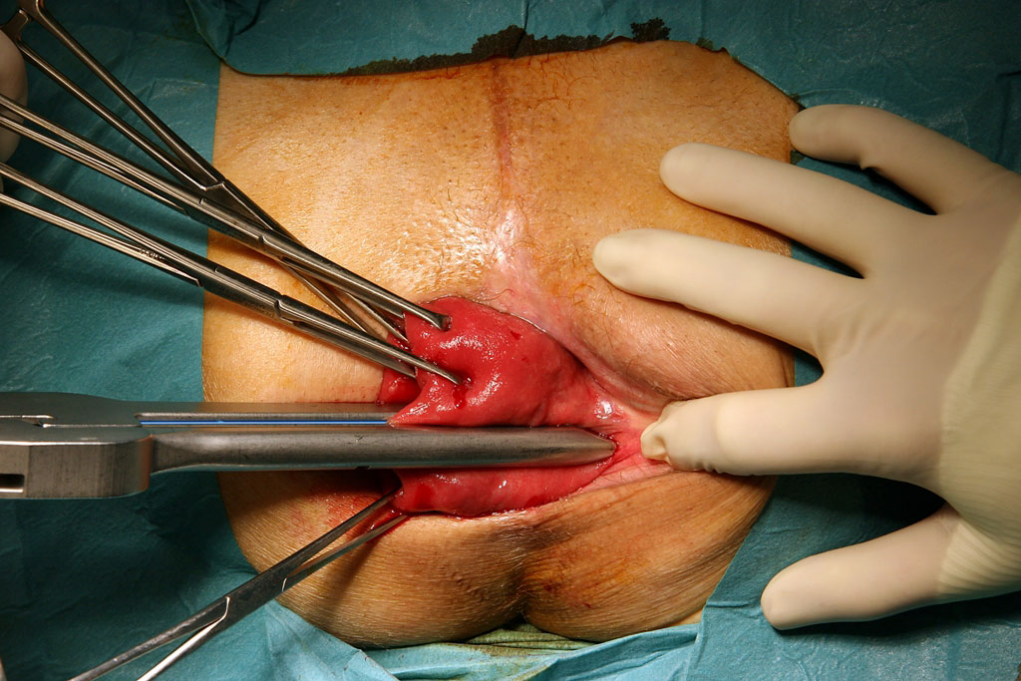
**Fixation**. The prolapse was completely pulled out and fixed by Allis clamps. Then the prolapse was axially cut open at three o'clock with a linear stapler.

**Figure 2:**
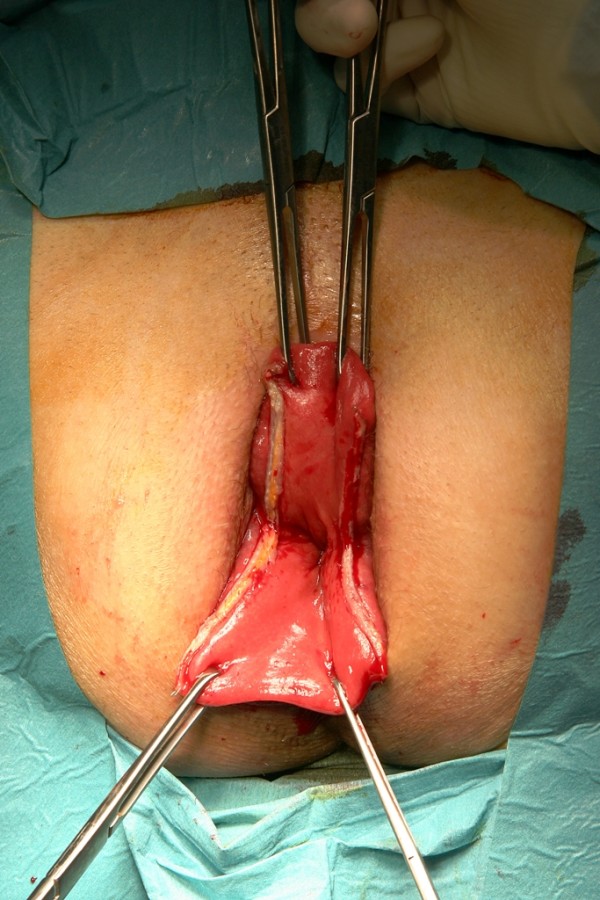
**Separation**. Separation of anterior and posterior wall of the prolapse after secondopening with a linear stapler at nine o'clock.

The surgical technique has been described in detail previously [1]; therefore, we present here only a short description and the main modifications. PSP was performed under spinal or general anaesthesia in a lithotomy position. To free the pouch of Douglas from any deep enterocele, a slight Trendelenburg position was chosen. The prolapse was completely pulled out and fixed by Allis clamps placed at its verge. To exclude the entrapment of any intraperitoneal organ in the prolapse, a very careful bi-manual examination was performed. In addition to the initial technique, the prolapse was axially cut open at the three (Fig. 1) and nine o'clock positions with a linear stapler (e.g., ILATM 100; US Surgical, Norwalk, CT, or Proximate^® ^Linear cutter 75 mm, Ethicon Endo-Surgery) (Fig. 2). The staple line ended 1 to 2 cm from the dentate line on both sides. To reach the described end of the opening cut, it is advisable to remove the white distal tip of the linear stapler on the anvil side and to introduce the linear stapler with the cartridge side into the prolapse. Doing this, the instrument is able to cut and staple just to the end of the anvil side under the surgeon's vision. In female patients, the stapler was fired after the digital exploration of the back wall of the vagina to exclude its entrapment.

**Figure 3:**
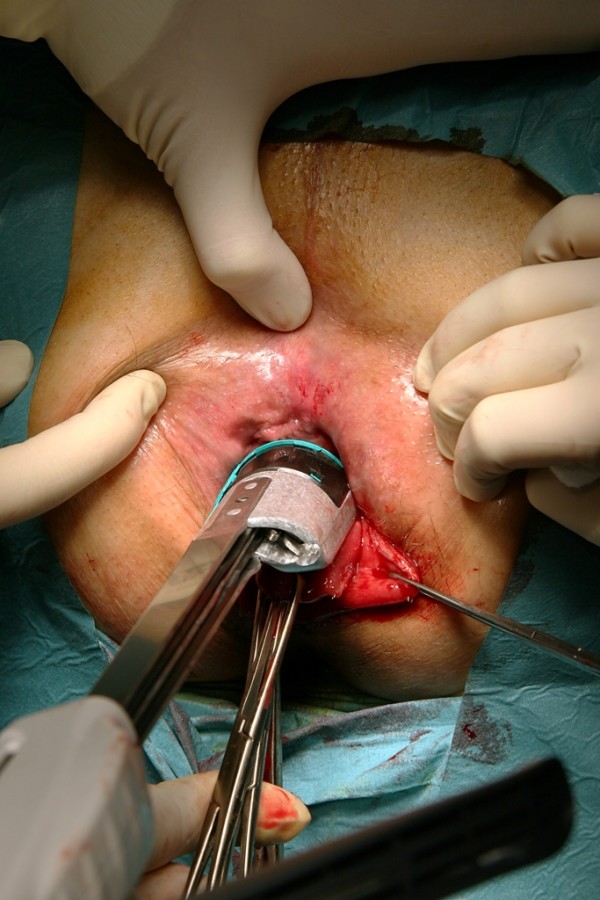
**Resection**. Resection of the prolapse continuously counterclockwise by the curved Contour^® ^Transtar^TM^ and parallel to the dentate line, first anteriorly.

The prolapse was resected continuously counterclockwise by the curved Contour^® ^TranstarTM and parallel to the dentate line, first anteriorly starting at three o'clock position (Fig. 3), second posteriorly beginning at nine o'clock. After completing the resection, the anal mucosa and the neorectum fell back into place spontaneously. The stapled resection line was inspected using a transparent speculum. Absorbable monofilament sutures were performed to ensure haemostasis and to strengthen the anastomosis. The weight of resected tissue was documented.

### Statistics

Median and range are shown for all data. Wexner score pre- and post-PSP were compared by the Wilcoxon matched pairs test. P < 0.05 was considered statistically significant.

## Results

**Figure 4:**
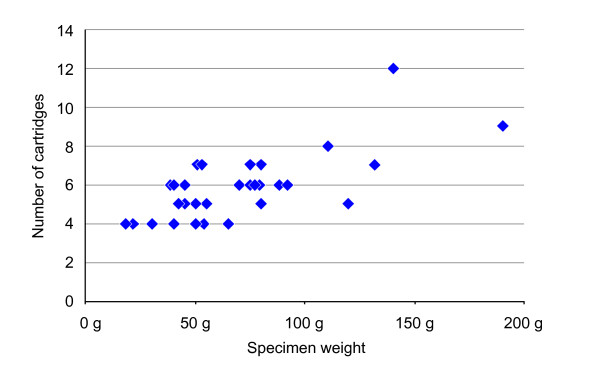
**Cartridges**. Number of cartridges used in correlation with the specimen weight.

**Figure 5:**
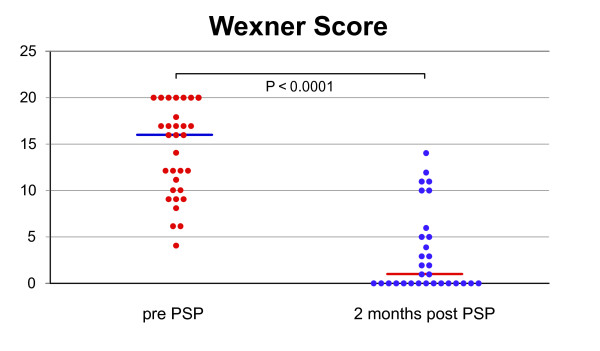
**Wexner faecal incontinence score**. (continent = 0, most severe incontinence = 20) before (= Pre-PSP) and after stapled perineal prolapse resection (PSP) (= Post-PSP). Horizontal line = median.

Thirty-nine consecutive patients with an external rectal prolapse were evaluated for surgical treatment. Seven patients opted for an abdominal procedure: 3 for a laparoscopic resection rectopexy, 2 for a laparoscopic suture rectopexy and 2 for a laparoscopic ventral rectopexy. The PSP was performed on 32 patients, with a median age of 80 years (range 26-93). Two of the patients were male. In 26 patients the external rectal prolapse was a first-time occurrence, whereas the other 6 patients suffered a recurrence and had undergone surgery at least once before using different techniques. The median American Society of Anaesthesiologists Grade was II (range I-III). Sixteen patients were operated on using spinal, the other sixteen using general anaesthesia. In all 32 patients, the PSP was successfully completed with no intraoperative complications. The median operation time was 30 minutes (range 15-65) and 6 cartridges(range 4-12) were used for the resection. The median weight of the specimen was 60 g (range 18-190). The number of cartridges used increased with the weight of the specimen (Fig. 4). The double axially stapled opening of the prolapse was performed on half of the patients. Complications occurred in 2 patients (6.3%) postoperatively within 30 days. One patient had a significant elevated body temperature of 38.5°C 17 days after the surgical intervention, caused by a common cold (complication grade I needing no further intervention) [7]. One patient with continuous ambulatory peritoneal dialysis (CAPD) developed peritonitis two days after the procedure and had to be treated for 14 days with antibiotics, grade II (complication needing medication) [7]. The median postoperative hospitalisation was 5 days (range 2-19). In one female patient functional data were not available. The median Wexner incontinence score before PSP was 16 (4-20), after PSP 1 (0-14), respectively (P < 0.0001) (Fig. 5). The median follow-up was 6 months (4-22). Before surgery twelve (39%) patients complained of constipation, 10 (31%) reported a continuation of their symptoms after surgery.

## Discussion

The excellent short-term outcome, described in the feasibility study on the PSP, could be successfully reproduced in this study with 32 patients. Additionally, the modified technique is faster and simpler and was performed without major complications. The considerable improvement in the patients' faecal continence after PSP and the functional results achieved confirm the advantages of PSP compared to established surgical techniques. Severe faecal incontinence disappeared in 90% of patients after PSP with no new onset of severe constipation occurring.

The more invasive a surgical procedure is, the longer the convalescence and the higher the complication rates can be [8]. Abdominal approaches have low recurrence rates but are associated with higher rates of morbidity and mortality [9]. One of the abdominal procedures is the mesh rectopexy with anterior or posterior insertion of foreign material such as fascia lata, non-absorbable synthetic meshes or absorbable meshes. The assumption is that this material evokes more fibrous tissue formation than an ordinary suture rectopexy [10]. The disadvantage of the posterior procedure is an increased risk of infectious complications, such as significant pelvic sepsis, which has been reported in 2% to 16% [11] of cases. In the event of a synchronous resection, the theoretical risk of infection is increased [12]. For the anterior sling rectopexy, called the Ripstein procedure, a 52% complication rate was determined by Roberts et al. in 1988. The presacral haematoma dominated with 8% of cases as the most frequent postoperative complication [13]. In 2000 Kellokumpu et al. compared the laparoscopic technique with laparotomy and demonstrated a reduction in postoperative pain and hospitalisation in the case of the laparoscopic rectopexy [14]. In spite of the advantages, the mortality for the laparoscopic technique still ranged between 0% and 3% [11].

For elderly and frail patients with co-morbidities, the perineal procedures such as the Delorme operation and the perineal rectosigmoidectomy represent low-risk techniques associated with high recurrence rates. In 2003 Watkins et al. published a long-term follow-up of a modified Delorme procedure with 52 patients. In 25% they observed postoperative complications, such as urinary retention, fever, hypokalaemia, cardiac arrhythmia, suture line dehiscence, perineal cellulitis and bleeding, according to grades I to IIIb of the Dindo classification [7]. Kimmins et al. presented their results of a series of 63 consecutive Altemeier repairs in 2001 [9]. Overall, they found anastomotic leak, stenosis, rectovaginal fistula and bleeding as postoperative complications in 11%. In the present study only two patients had an unacceptable postoperative follow-up (6.3%). In one patient with CAPD we had to treat a potential severe complication. Two days after the procedure she developed a temperature and abdominal pain and the dialysat presented signs of peritonitis. The abdominal CT scan showed an air bubble on the anastomosis side and signs of inflamed tissue. Fortunately, this small anastomosis leakage and peritonitis was successfully treated with i.v. antibiotics and parenteral nutrition for 10 days while the patient continued peritoneal dialysis. We recommend a careful over-suturing of the stapler line to prevent postoperative bleeding and to minimise anastomosis dehiscence. It is worthy of note that the open techniques are accompanied by a mortality rate of 0-16% compared to other perineal procedures such as the Delorme operation with 4-38% and Altemeier's rectosigmoidectomy with 0-16%. Fortunately, we had no mortality in our study of PSP.

In our experience, the ideal patients for PSP are those with a rectal prolapse, maximum length of 10 cm and a weight of up to 100 g. Larger prolapses, such as a colon sigmoideum prolapse, are technically more demanding, increasing the use of cartridges and the duration of the operation (Fig. 4).

A part of the cure of the prolapse, the most important fact for the patient, is the functional outcome of the surgical treatment chosen. In the following are the three most determinant aspects of functional outcome: incontinence, constipation and the consequences of nerve damage and adhesions.

### Incontinence

The total rectal prolapse is accompanied by faecal incontinence ranging from 35-100% [15]. In the present study, 21 of 31 patients (68%) complained of preoperative faecal incontinence. After the PSP the faecal incontinence improved considerably in 9 patients or even disappeared completely in 15 patients (Fig. 5). In two patients (6.5%), one with low grade faecal incontinence and the other with no faecal incontinence, the PSP worsened the situation. One of them complained of massive preoperative constipation and was very satisfied about the altered situation. The improvement in continence after PSP is in accordance with other surgical techniques for rectal prolapse. Except for a few particular cases, all current surgical procedures improve faecal continence. In 1996 Graf et al. described a worsening of continence in 12% after suture rectopexy in a retrospective study with 53 patients [16]. In 1982 Launer et al. presented a retrospective study with 54 patients [17] and Schultz et al. published a study with 69 patients in 2000 after the Ripstein procedure [18]. Both determined a 10% worsening of the preoperative incontinence as well as declared after rectopexy.

### Constipation

One of the salient postoperative complications of the transabdominal surgical techniques is constipation up to 50% [19]. In particular, existing preoperative constipation might worsen after the transabdominal intervention [20-22]. Some authors recommend a concomitant resection [23,21,27], which decreases the postoperative constipation rate by about 50% [28], but unfortunately increases the mortality rate to 10-15% because of the potential insufficiency of the anastomosis. None of the patients complained postoperatively of a renewed onset of constipation during the follow-up period. However, the follow-up period is too short to evaluate patients according to the preoperative criteria.

### Nerve damage and adhesion

The high risk of sexual dysfunction for men and the dysfunction of micturition after transabdominal rectopexy is caused by nerve damage. In 1998 Yakut et al. declared that the risk for impotence for men after transabdominal rectopexy with dorsal mobilisation is 17% [29]. A modified technique was introduced by D'Hoore et al. In 2004 [19] and two years later the author presented a study with 109 patients. All underwent the new, autonomic nerve-sparing laparoscopic technique, called laparoscopic ventral recto(colpo)pexy for rectal prolapse [30]. The aim of this study was to assess the mid-term outcome with regard to functional outcome, morbidity and recurrence rate. Although no mortality or major morbidity occurred and minor morbidity was noted for 7% of the patients, and a recurrence rate of 3.7% was observed, the risks mentioned below of intrabdominal surgical techniques should not be discounted. Every transabdominal surgery causes adhesions. For women of childbearing age these adhesions might result in infertility. For that reason one female patient of 27 years preferred the PSP, and another because of continuous ambulatory peritoneal dialysis.

An early recurrence was observed in one patient. She had been operated on using the Delorme technique twice before and the rectal prolapse occurred 11 months after the PSP intervention. We resolved the recurrence with a laparoscopic ventral rectopexy. The data provided represent only a mid-term follow-up and a comparison to other surgical techniques is not reasonable.

In the follow-up interview we asked every patient about his satisfaction with the result after the surgical intervention. Twenty-four (77%) patients spontaneously assessed the PSP as the right decision and were content to very content with the outcome.

## Conclusion

The present study confirms the good subjective and objective results of the previous feasibility study [1]. The modified technique with the two axially stapled cuts at three and nine o'clock simplifies and accelerates the intervention and allows it to be safely performed. The results and the recurrence rate of the present mid-term follow-up are comparable or even superior to those of current perineal methods and encourage taking the indication of PSP beyond the elderly and frail patient. One limitation is the unknown recurrence rate in the long-term follow-up; however, if necessary a recurrence can be successfully treated by an abdominal procedure.

## Competing interests

Franc H. Hetzer is consultant of Ethicon Endo-Surgery for stapled transanal rectal resection (STARR) with Contour Transtar, but not for the Perineal Stapled Prolapse resection (PSP) technique presented in the manuscript.

## Authors' contributions

FHH: conceived the study, performed the surgery, participated in data acquisition and prepared the manuscript. AR: participated in interpretation of data, draft revision and data acquisition. KW: participated in draft revision and data acquisition. UB: performed data management and analysis, prepared table and figure drafts, participated in draft revision.  JL: participated in study design and draft revision. LM: participated in draft revision and interpretation of data, performed the surgery. All authors read and approved the final manuscript

## Pre-publication history

The pre-publication history for this paper can be accessed here:

http://www.biomedcentral.com/1471-2482/10/9/prepub

## References

[B1] Scherer R Marti L Hetzer FH Perineal stapled prolapse resection: a new procedure for external rectal prolapse Dis Colon Rectum 2008 51 1727 1730 10.1007/s10350-008-9423-0 18626711

[B2] Mikulicz J Zur operativen Behandlung des Prolapsus recti et coli invaginati Langenbecks Arch Chir 1889 38 74 97

[B3] Agachan F Reissman P Pfeifer J Weiss EG Nogueras JJ Wexner SD Comparison of three perineal procedures for the treatment of rectal prolapse South Med J 1997 90 925 932 9305305 10.1097/00007611-199709000-00013

[B4] Azimuddin K Khubchandani IT Rosen L Stasik JJ Riether RD Reed JF Rectal prolapse: a search for the "best" operation Am Surg 2001 67 622 627 11450773

[B5] Hoel AT Skarstein A Ovrebo KK Prolapse of the rectum, long-term results of surgical treatment Int J Colorectal Dis 2009 24 201 207 10.1007/s00384-008-0581-2 18791726

[B6] Drossman DA The Rome Foundation and Rome III Neurogastroenterol Motil 2007 19 783 786 10.1111/j.1365-2982.2007.01001.x 17883428

[B7] Dindo D Demartines N Clavien PA Classification of surgical complications: a new proposal with evaluation in a cohort of 6336 patients and results of a survey Ann Surg 2004 240 205 213 10.1097/01.sla.0000133083.54934.ae 15273542 PMC1360123

[B8] Agachan F Pfeifer J Joo JS Nogueras JJ Weiss EG Wexner SD Results of perineal procedures for the treatment of rectal prolapse Am Surg 1997 63 9 12 8985063

[B9] Kimmins MH Evetts BK Isler J Billingham R The Altemeier repair: outpatient treatment of rectal prolapse Dis Colon Rectum 2001 44 565 570 10.1007/BF02234330 11330584

[B10] Kuijpers HC Treatment of complete rectal prolapse: to narrow, to wrap, to suspend, to fix, to encircle, to plicate or to resect? World J Surg 1992 16 826 830 10.1007/BF02066977 1462615

[B11] Madiba TE Baig MK Wexner SD Surgical management of rectal prolapse Arch Surg 2005 140 63 73 10.1001/archsurg.140.1.63 15655208

[B12] Athanasiadis S Weyand G Heiligers J Heumuller L Barthelmes L The risk of infection of three synthetic materials used in rectopexy with or without colonic resection for rectal prolapse Colorectal Dis 1996 11 42 44 10.1007/BF00418855 8919341

[B13] Roberts PL Schoetz DJ Coller JA Veidenheimer MC Ripstein procedure. Lahey Clinic experience: 1963-1985 Arch Surg 1988 123 554 557 3358680 10.1001/archsurg.1988.01400290036005

[B14] Kellokumpu IH Vironen J Scheinin T Laparoscopic repair of rectal prolapse: a prospective study evaluating surgical outcome and changes in symptoms and bowel function Surg Endosc 2000 14 634 640 10.1007/s004640000017 10948299

[B15] Zbar AP Takashima S Hasegawa T Kitabayashi K Perineal rectosigmoidectomy (Altemeier's procedure): a review of physiology, technique and outcome Tech Coloproctol 2002 6 109 116 10.1007/s101510200024 12402057

[B16] Graf W Karlbom U Pahlman L Nilsson S Ejerblad S Functional results after abdominal suture rectopexy for rectal prolapse or intussusception Eur J Surg 1996 162 905 911 8956961

[B17] Launer DP Fazio VW Weakley FL Turnhull RB Jagelman DG Lavery DP The Ripstein procedure: a 16-year experience Dis Colon Rectum 1982 25 41 45 10.1007/BF02553547 7056140

[B18] Schultz I Mellgren A Dolk A Johansson C Holmstrom B Long-term results and functional outcome after Ripstein rectopexy Dis Colon Rectum 2000 43 35 43 10.1007/BF02237241 10813121

[B19] D'Hoore A Cadoni R Penninckx F Long-term outcome of laparoscopic ventral rectopexy for total rectal prolapse Br J Surg 2004 91 1500 1505 10.1002/bjs.4779 15499644

[B20] Aitola PT Hiltunen KM Matikainen MJ Functional results of operative treatment of rectal prolapse over an 11-year period: emphasis on transabdominal approach Dis Colon Rectum 1999 42 655 660 10.1007/BF02234145 10344689

[B21] Salkeld G Bagia M Solomon M Economic impact of laparoscopic versus open abdominal rectopexy Br J Surg 2004 91 1188 1191 10.1002/bjs.4643 15449272

[B22] Schultz I Mellgren A Oberg M Dolk A Holmstrom B Whole gut transit is prolonged after Ripstein rectopexy Eur J Surg 1999 165 242 247 10.1080/110241599750007117 10231658

[B23] Huber JP Gregorcyk S Anorectal Disease Curr Treat Options Gastroenterol 2000 3 229 242 10.1007/s11938-000-0026-7 11097740

[B24] Johnson E Stangeland A Johannessen HO Carlsen E Resection rectopexy for external rectal prolapse reduces constipation and anal incontinence Scand J Surg 2007 96 56 61 17461314 10.1177/145749690709600111

[B25] Madoff RD Williams JG Wong WD Long-term functional results of colon resection and rectopexy for overt rectal prolapse Am J Gastroenterol 1992 87 101 104 1728105

[B26] Sayfan J Pinho M Alexander-Williams J Keighley MR Sutured posterior abdominal rectopexy with sigmoidectomy compared with Marlex rectopexy for rectal prolapse Br J Surg 1990 77 143 145 10.1002/bjs.1800770209 2317672

[B27] Schlinkert RT Beart RW Jr Wolff BG Pemberton JH Anterior resection for complete rectal prolapse Dis Colon Rectum 1985 28 409 412 10.1007/BF02560226 4006636

[B28] Benoist S Taffinder N Gould S Chang A Darzi A Functional results two years after laparoscopic rectopexy Am J Surg 2001 182 168 173 10.1016/S0002-9610(01)00672-9 11574090

[B29] Yakut M Kaymakcioglu N Simsek A Tan A Sen D Surgical treatment of rectal prolapse. A retrospective analysis of 94 cases Int Surg 1998 83 53 55 9706519

[B30] D'Hoore A Penninckx F Laparoscopic ventral recto(colpo)pexy for rectal prolapse: surgical technique and outcome for 109 patients Surg Endosc 2006 20 1919 1923 10.1007/s00464-005-0485-y 17031741

